# Study protocol: a pragmatic, cluster-randomized controlled trial to evaluate the effect of implementation of the Truenat platform/MTB assays at primary health care clinics in Mozambique and Tanzania (TB-CAPT CORE)

**DOI:** 10.1186/s12879-023-08876-8

**Published:** 2024-01-19

**Authors:** V. N. Leukes, J. Hella, I. Sabi, M. Cossa, C. Khosa, B. Erkosar, C. Mangu, E. Siyame, B. Mtafya, A. Lwilla, S. Viegas, C. Madeira, A. Machiana, J. Ribeiro, A. L. Garcia-Basteiro, F. Riess, D. Elísio, M. Sasamalo, G. Mhalu, C. M. Denkinger, M. D. M. Castro, S. Bashir, S. G. Schumacher, E. Tagliani, A. Malhotra, D. Dowdy, C. Schacht, J. Buech, D. Nguenha, N. Ntinginya, M. Ruhwald, A. Penn-Nicholson, K. Kranzer, TB-CAPT Consortium

**Affiliations:** 1 FIND, Geneva, Switzerland; 2 Ifakara Health Institute, Dar Es Salaam, Tanzania; 3 Mbeya Medical Research Centre, National Institute for Medical Research (NIMR), Mbeya, Tanzania; 4 Centro de Investigação Em Saúde de Manhiça (CISM), Manhica, Mozambique; 5 Instituto Nacional de Saúde (INS), Marracuene, Mozambique; 6 ISGlobal, Hospital Clínic – Universitat de Barcelona, Barcelona, Spain; 7 Division of Infectious Diseases and Tropical Medicine, Ludwig Maximilian University Hospital, Munich, Germany; 8 Division of Infectious Disease and Tropical Medicine and German Centre for Infection Research, Heidelberg University Hospital, Heidelberg, Germany; 9 Emerging Bacterial Pathogens Unit, IRCCS San Raffaele Scientific Institute, Milan, Italy; 10 Johns Hopkins University (JHU), Baltimore, MD USA; 11 LINQ Management, Berlin, Germany; 12 Clinical Research Department, London School of Hygiene and Tropical Medicine, London, UK

**Keywords:** Tuberculosis, Diagnostics, Truenat, Implementation, Efficacy, Clinical trial

## Abstract

**Background:**

In 2020, the WHO-approved Molbio Truenat platform and MTB assays to detect *Mycobacterium tuberculosis* complex (*MTB*) and resistance to rifampicin directly on sputum specimens. This primary health care center-based trial in Mozambique and Tanzania investigates the effect of Truenat platform/MTB assays (intervention arm) combined with rapid communication of results compared to standard of care on TB diagnosis and treatment initiation for microbiologically confirmed TB at 7 days from enrolment.

**Methods:**

The Tuberculosis Close the Gap, Increase Access, and Provide Adequate Therapy (TB-CAPT) CORE trial employs a pragmatic cluster randomized controlled design to evaluate the impact of a streamlined strategy for delivery of Truenat platform/MTB assays testing at primary health centers. Twenty-nine centers equipped with TB microscopy units were selected to participate in the trial. Among them, fifteen health centers were randomized to the intervention arm (which involves onsite molecular testing using Truenat platform/MTB assays, process process optimization to enable same-day TB diagnosis and treatment initiation, and feedback on Molbio platform performance) or the control arm (which follows routine care, including on-site sputum smear microscopy and the referral of sputum samples to off-site Xpert testing sites). The primary outcome of the study is the absolute number and proportion of participants with TB microbiological confirmation starting TB treatment within 7 days of their first visit. Secondary outcomes include time to bacteriological confirmation, health outcomes up to 60 days from first visit, as well as user preferences, direct cost, and productivity analyses.

**Ethics and dissemination:**

TB-CAPT CORE trial has been approved by regulatory and ethical committees in Mozambique and Tanzania, as well as by each partner organization. Consent is informed and voluntary, and confidentiality of participants is maintained throughout. Study findings will be presented at scientific conferences and published in peer-reviewed international journals.

**Trial Registration:**

US National Institutes of Health’s ClinicalTrials.gov, NCT04568954. Registered 23 September 2020.

**Supplementary Information:**

The online version contains supplementary material available at 10.1186/s12879-023-08876-8.

## Background

Timely and appropriate diagnosis and treatment is the key to reduce tuberculosis (TB) mortality, morbidity and prevent transmission. However, 40% (4.2 million) of the 10.6 million people with TB and two-thirds of those with multi-drug resistant (MDR) TB remain undiagnosed [1]. The main reasons are inaccessibility of diagnostics and attrition during the diagnostic process [2–5]. Improved systematic delivery and access to quality diagnostic testing, at healthcare centers closer to patients’ homes would have a significant impact in decreasing the “missing millions” who die due to undiagnosed TB or are diagnosed very late, resulting in severe and irreversible lung damage [6].

Bottlenecks at various points of the diagnostic pathway result in substantial attrition during the diagnostic process. Essential steps to diagnosis include the individual accessing care, the health care worker referring the individual for TB investigations, the specimen being sent for TB diagnostics and the results being returned. Initiation of appropriate treatment relies on timely receipt of laboratory results and the patient returning to clinic. Many Sub-Saharan countries have reported these bottlenecks along their diagnostic pathway [7–10].

Although the Xpert MTB/RIF and Xpert MTB/RIF Ultra (“Ultra”) operated on the GeneXpert platform was a game-changer in the diagnosis of both TB and rifampicin (RIF) resistance, endorsed by the WHO in 2011 and 2017, respectively, its roll-out in many countries remains a centralized service [8, 11]. Global financial constraints and the COVID-19 pandemic demonstrated that countries heavily reliant on a single diagnostic solution for TB such as Xpert, were more susceptible to stock outs and testing restrictions for high-risk groups [12, 13]. Additionally, across Africa, TB notifications have not increased since the introduction of Xpert and the impact on mortality remains uncertain [14]. To address some of the limitations experienced during Xpert rollout, decentralized, rapid, accurate molecular diagnostic assays, from a diversified pipeline of diagnostics developers are required [15, 16].

The Molbio Truenat platform designed to be operated as a near point-of-care diagnostic solution in peripheral laboratories or primary health clinics with minimal infrastructure, was endorsed in 2020 by the WHO for TB diagnosis. The platform consisting of the Trueprep DNA extraction device and the Truelab micro-PCR machine runs the Truenat MTB Plus assay for detection of *Mycobacterium tuberculosis* complex (MTBC) and Truenat MTB-RIF Dx for detection of RIF resistance (collectively referred to as the Truenat MTB assays). Operationally, it takes 20 min to do the DNA extraction and another 35 min to diagnose TB. If a test is positive for TB, extracted DNA eluate from the initial sample can be used for RIF-resistance reflex testing. The limit of detection is estimated to be 100 colony forming units (CFU)/ml in sputum sample, similar to that of Xpert MTB/RIF. When trialed at primary health clinics in high TB incidence countries, sensitivity was 80% (95% CI 75–84%) and specificity was 96% (95% CI 95–97%) for Truenat MTB Plus, as compared to a reference standard of any culture-positive result across 2 × sputa collected on consecutive days. At the primary health clinic, the Truenat MTB-RIF Dx assay had 84% (95% CI 62–95%) sensitivity and 95% (95% CI 90–97%) specificity for RIF resistance detection. In head-to-head comparisons with both Xpert MTB/RIF or Ultra, there was no difference in performance of Truenat MTB assays [17]. While performance data of the Truenat MTB assays in decentralised settings are promising, this does not automatically translate into better patient-important outcomes. Especially when near patient testing is introduced as part of routine clinical care rather than implemented under well controlled research conditions.

The TB-CAPT CORE trial has been designed to assess the effectiveness, user acceptability and cost effectiveness of the Truenat platform/MTB assays in improving the TB diagnostic pathway in primary health clinics in Mozambique and Tanzania using a pragmatic design.

## Methods

### Study objectives

TB-CAPT CORE’s primary objective is to evaluate the effect of near point-of-care testing for TB using the Truenat platform/MTB assays in primary health clinics combined with rapid communication (intervention arm), compared to the current standard of care of offsite Xpert testing (control arm) on TB diagnosis and treatment initiation for microbiologically confirmed TB at 7 days from enrolment. The secondary objectives are to evaluate the effect of the Truenat platform/MTB assays on time to TB treatment initiation at 60 days from enrolment for microbiologically confirmed TB cases, clinically diagnosed TB cases and all TB cases (clinically diagnosed and microbiologically confirmed); estimate the effect of the Truenat platform/MTB assays on morbidity, mortality, on treatment loss to follow-up at 7 and 60 days from enrolment; estimate the effect of Truenat platform/MTB assays on patient costs related to care; and evaluate the reliability of Truenat platform/MTB assays as measured by rate of non-determined test results and platform failure.

Additionally, user preferences will be evaluated by (1) Investigating user perspectives on the Truenat platform/MTB assays (including perspectives of end-users such as patients, but also of professional users such as laboratory technicians, clinicians, nurses, and decision-makers) for use as a diagnostic test for outpatients presenting with symptoms suggestive of pulmonary TB, (2) Understanding experiences and challenges with TB diagnostic testing using different diagnostic approaches (Xpert® MTB/RIF, Xpert® MTB/RIF Ultra, microscopy, culture) (3) Examining feasibility and preferences with regard to diagnosing TB and how Truenat platform/MTB assays change these, and (4) Assessing the usability and acceptability of Truenat platform/MTB assays in the intended users.

### Study endpoints

**Table:** 

Primary	Absolute number and point estimate (with 95% confidence intervals (CIs)) of the proportion of enrolled participants who are diagnosed with microbiologically confirmed TB and are starting TB treatment within 7 days of enrolment
Secondary	
-Diagnosis	Time to bacteriological confirmation of TB (up to 60 days) from enrolment and point estimate with 95% CIs of the proportion of patients treated for TB who are diagnosed up to 60 days from enrolment (microbiologically and/or clinically)
-Treatment	Point estimate with 95% CIs of the proportion of participants evaluated for pulmonary TB starting TB treatment with microbiological confirmation within 60 days from enrolment
	Absolute number, and point estimate (with 95% CIs) of the proportion of enrolled participants evaluated for pulmonary TB starting TB treatment regardless of microbiological confirmation within 7 and 60 days from enrolment
	Time to TB treatment initiation for those with microbiological confirmation and for all participants (censored at 60 days) from enrolment
	Point estimate of the proportion of patients with ongoing treatment
-Morbidity	Prevalence of current cough, limited appetite, weakness at 60 days from enrolment
-Cost effectiveness	Estimate of patient costs related to care, number of lost working days, monthly earnings, unit costs of diagnosis and treatment, incremental cost-effectiveness ratio
-Operational characteristics	Proportion of Invalid/Error Truenat MTB-Assay test results and platform failure
-User acceptability	User survey to assess usability

### Intervention

The intervention is the placement of the Truenat platform/MTB assays at primary health clinics combined with rapid communication of results and same day TB treatment initiation. In addition, laboratory technicians who are responsible for the smear microscopy were trained on the use of the Truenat platform/MTB assays.

The standard of care is either on-site smear microscopy and/or off-site Xpert MTB/RIF testing.

### Study design

TB-CAPT CORE is a pragmatic cluster randomized controlled trial enrolling adults presenting to primary health clinics with symptoms suggestive of pulmonary TB. Clinics randomized to the intervention arm have the Truenat platforms introduced at the facility and sputum samples undergo testing with Truenat MTB Plus and, if positive, extracted DNA is reflexed to the Truenat MTB-RIF Dx assay. Adults accessing clinics randomized to standard of care are investigated according to the national standard, which in most cases is off-site Xpert testing and/or smear microscopy. In addition, procedures are put in place to ensure rapid communication of results and possibly same-day TB treatment initiation in the intervention clinics.

Eligibility criteria for recruitment into the study included: i) presentation to one of the trial clinics with symptoms suggestive of pulmonary TB as defined by the national TB treatment guidelines in each country [18, 19], including cough for more than 1–2 weeks and/or fever, night sweats, blood-stained sputum (haemoptysis) significant weight loss, abnormalities on chest radiograph ii) able to provide a sputum sample iii) ≥ 18 years and iv) able and willing to consent.

Participants are excluded if there are circumstances that raise doubt of free, informed consent (e.g., in a mentally impaired person or a prisoner); already diagnosed with TB; currently receiving anti-TB therapy; who are seriously ill and need to be admitted to hospital; or have been enrolled into the trial at a previous visit.

### Recruitment sites

**Fig. 1:**
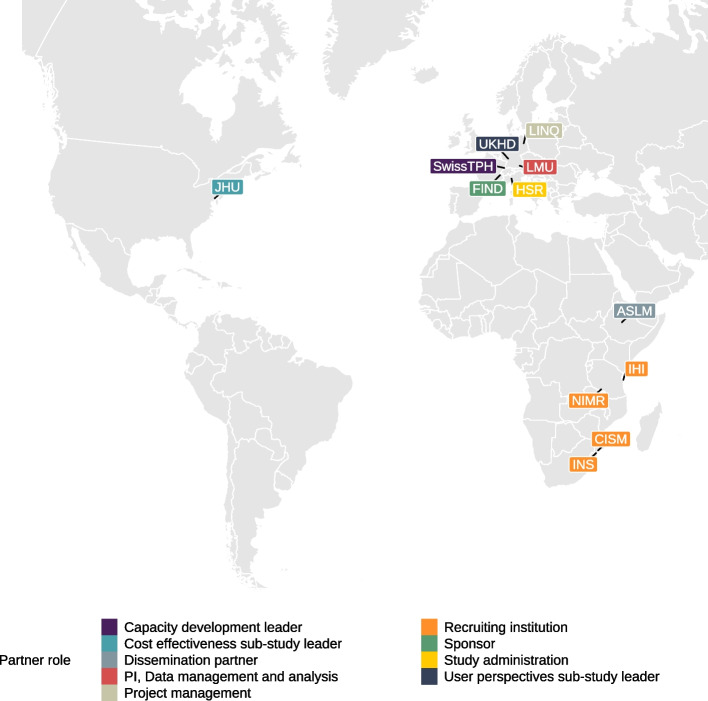
TB-CAPT consortium partners contributing to the TB-CAPT CORE trial

**Fig. 2:**
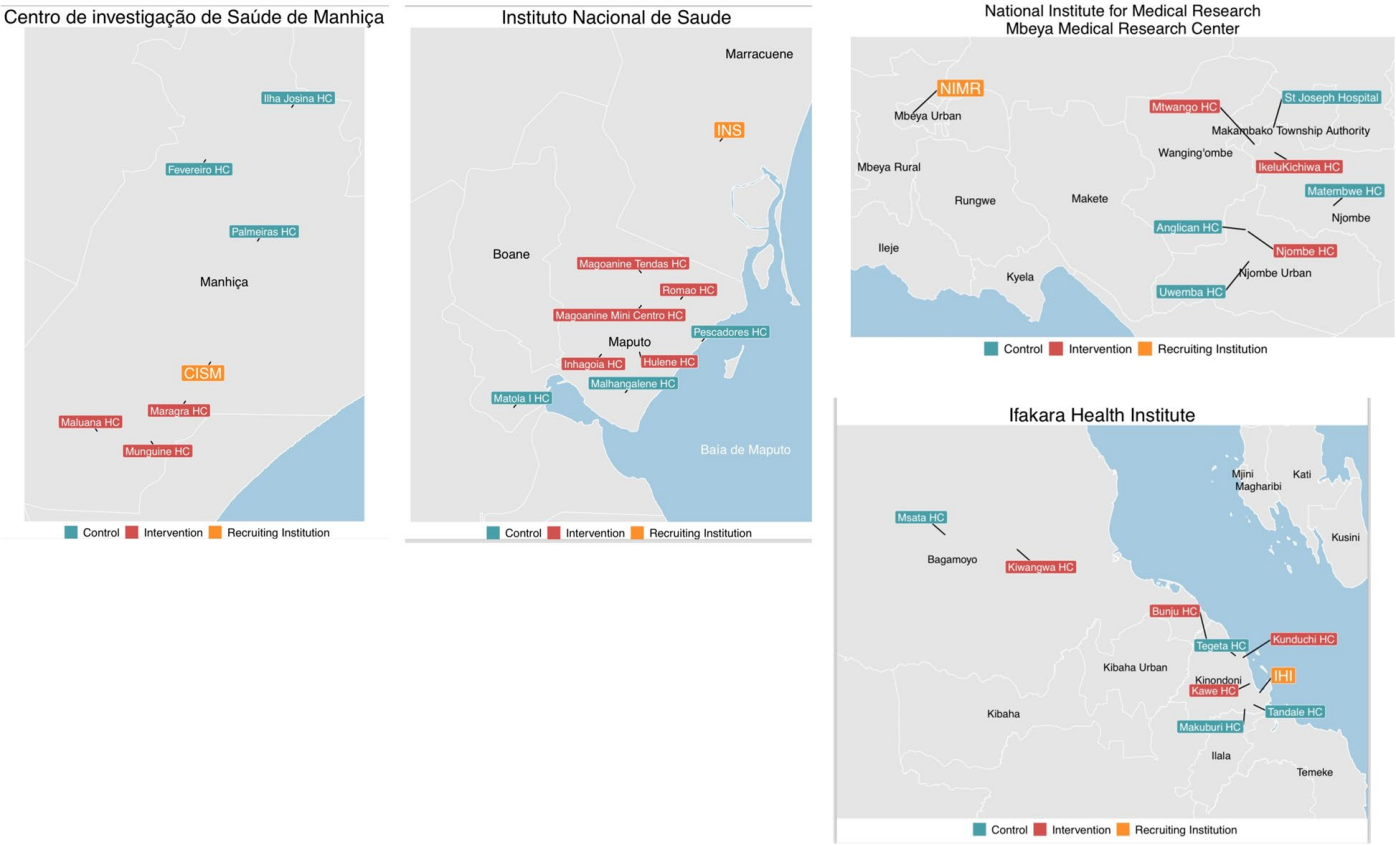
Geographical locations of Recruiting institutions and their associated primary health centers. Clinics assigned to the control and intervention arm are indicated in blue and red, respectively. **A** Centro de investigação de Saúde de Manhiça (CISM), Manhica, Mozambique. **B** Instituto Nacional de Saúde (INS), Maputo, Mozambique. **C** The National Institute for Medical Research (NIMR), Mbeya, Tanzania. **D** Ifakara Health Institute (IHI), Dar es Salaam, Tanzania

Partners of the TB-CAPT consortium and their role within the trial are indicated in Fig. 1. Trial clinics are located in the city of Maputo and the district of Manhica in Mozambique and Dar es Salaam and Njombe in Tanzania (Fig. 2). Recruitment commenced in August 2022 at all sites.

### Randomization of clinics

The unit of randomization in this trial is a clinic. Before randomization, all four African research institutions were asked to identify clinics that offered TB treatment, did not have a GeneXpert laboratory on-site and were willing to participate in the trial. Selection of eligible clinics was discussed and agreed upon with the respective National TB Programmes, Potential trial clinics were asked to provide quarterly TB notification data covering the period January 2018- December 2019.

From this information the estimated number of people presenting with symptoms of pulmonary TB (“size of a clinic”) were derived. This information was used as a strata variable in the randomization process. The clinics were randomly assigned to Truenat platform/MTB assays (intervention) or standard of care (control) arms following a restricted randomization strategy [20], whereby 6–8 strata of clinics (‘clusters’) were established using the stratification variables; site (collaborating research institution) and size and applying balancing criteria for them. The number of strata per site was informed by the heterogeneity of the sizes of clinics, typically 1 or 2 strata per site were established.

A total of 29 clinics were randomized at the four participating institutions aiming to enroll up to 150 adults (≥ 18 years of age) each (total sample size 4200) with signs and symptoms suggestive of TB. Provision was made to increase the number of clinics (or replace clinics) to a maximum of 36 dependent on recruitment rates, clinic closures (due to COVID19) and drop out of clinics due to placements of GeneXpert instruments on-site.

### Sample size

Sample size calculations were performed for a matched cluster randomized trial with equal number of clusters in the control and intervention arms. Across intervention and control arm we assumed 12% TB prevalence and 10% loss to follow-up resulting in missing primary outcome information. With regards to the effect of the intervention we assumed a 20% higher pre-treatment loss to follow-up rate and/or delayed treatment start in the control compared to the intervention arm. Cluster size was set at 150 participants and the within-pair coefficient of variation was assumed to be 0.25. A sample size of 13 cluster pairs (3900 participants) would have provided 80% power to detect a difference between intervention and control arm (alpha-level 0.05). In order to allow for uncertainties, we included one more pair of clusters resulting in 28 clinics aiming to recruit 4200 participants. However, smaller average cluster sizes were observed during the set-up period if recruitment was limited to 6 months. Hence an additional 8 clinics were identified bringing the number of available clusters to 36. These additional clinics were also included in the randomization process to allow for inclusion of more clusters.

### Procedures

#### Main trial

Adults presenting with symptoms suggestive of pulmonary TB to one of the study clinics are invited to be screened for inclusion in the study. Following informed consent all participants are assigned a unique study identification number (Study ID) and a paper-based questionnaire is administered capturing sociodemographic data, information on duration and presence of symptoms, past medical history and household assets. A more detailed questionnaire to collect cost effectiveness data is administered to approximately every 10th participant, at each clinic. All participants are asked to provide contact details including their own or family phone number, address and the contact details of a trusted person who can be contacted if the participants cannot be reached.

At intervention clinics, microscopists were trained to use the Truenat platform, and to perform the Truenat assays before study initiation. Sputum specimens collected from participants at intervention clinics undergo Truenat MTB assays testing. Repeat testing is performed using the same DNA eluate if an invalid or error result is obtained. In case of inconclusive result on repeat testing, clinicians are informed, and a new sputum sample requested as per clinical indication. DNA eluates of samples testing positive for MTBC undergo MTB-RIF Dx assay reflex testing. If MTB RIF-Dx results are indeterminate or an error is obtained, clinicians are advised to ask the participant for a second sputum sample. Participants with positive MTB Plus results are referred for TB treatment initiation. Each institution and their linked clinics developed procedures to ensure rapid communication of Truenat platform/MTB assays results to clinicians. Participants are invited to remain at intervention clinics until Truenat TB testing has been completed to facilitate same-day treatment initiation.

At control clinics, staff received refresher training on the national guidelines on how to diagnose and treat TB. Sputum samples from participants at control clinics are investigated according to the standard procedures which includes smear microscopy on-site and/or Xpert testing off-site. Following sites assessment, several operational bottlenecks were identified such as stock outs of sputum containers and unpredictable sample transport to referral laboratories, which on many occasions led to referral of people rather than specimens to testing sites. Hence provisions have been made to ensure availability of sputum containers throughout the study period, and regular sample pick-ups at least twice per week.

Primary and secondary outcomes related to treatment, morbidity and mortality are determined by follow-up phone calls on day 7 and day 60. Three attempts are made to contact a participant by phone. If a participant cannot be reached, three attempts are made to contact the trusted person by phone and/or a home visit is performed. The contact window for day 7 and 60 interviews is 8–21-days and 61–90-days post-enrollment, respectively. If participants cannot be reached within the window of the 7-day call, new attempts to contact them are made at 60 days. TB results and TB treatment initiation data are extracted from clinic notes, laboratory, and TB registers. TB treatment status and start date are verified by comparing self-report and TB register information. Any discordance is resolved by contacting both the participant and the staff at the clinic.

#### User preferences sub-study

To assess user preferences, we use a qualitative approach with semi-structured interviews with patients, professional users (laboratory staff, nurses, clinicians) and decision makers. Direct observations of testing procedures and self-administered surveys are conducted to assess the usability of the Truenat platform/MTB assays.

Inclusion criteria for semi-structured interviews are a) age ≥ 18 years old and b) either: (1) Routine health workers participating in the CORE trial or (2) Patient tested or to be tested with the Truenat platform/MTB assays as part of the study or (3) Decision maker involved with implementation of novel tests at the local, national and/or regional level.

Eligible participants for the interviews and observations are invited to participate by study staff and, if they agree, sign the written informed consent form. Interviews are conducted in the participants’ preferred language, following a topic guide tailored to each group (patients, professional users, and decision makers). Topics for patients include experiences during the diagnostic process, preferences regarding sampling, time to results, availability of tests, distance to health facility and preferences to minimize issues related to distance, among others. In addition, for professional users and decision makers, topics regarding Truenat implementation, challenges, decentralization, current TB diagnosis algorithm, samples, etc. are explored. Duration of the interviews is approximately one hour.

Up to ten eligible health workers (operators of the test) per country at the intervention clinics are asked to perform specimen collection and/or testing under direct observation. After informed consent is obtained, a trained research staff records any errors or failures to complete any of the tasks required to operate the device. During these observations, health workers are also encouraged to provide general comments on device usability. Then they are asked to complete the System Usability Scale (SUS), a 10-item questionnaire on a 5-point Likert scale, to rate the usability of the test, and additional questions rate the ease-of-use of the Truenat platform /MTB assays [21, 22].

##### *Sample size and participant sampling for user preferences*

To gain insights from various professional users, 3–5 health workers from each user group (including nurses, clinicians and laboratory technicians involved in routine care provision as well as study staff involved in the study) are invited for an interview at each coordinating site. In addition, up to 3 TB and HIV programme officers (decision makers) are invited for an interview, which will allow us to gain insights in the perspective of decision makers in the program at country. In total, we seek to interview approximately 10–15 health care workers and decision makers per country.

To gain end-user perspectives (patients) on the Truenat platform/MTB assays for use as a diagnostic test for outpatients presenting with symptoms suggestive of pulmonary TB, we invite 10–12 participants from each coordinating site (approx. 20–24 per country) for an interview, either when being enrolled in the study or later through the follow-up mechanisms that are part of the study. We will aim to obtain an equal number of patients who tested positive or negative, as well as from the intervention and control clinics.

Sample size determination in qualitative research rests on maximizing potential for “saturation” (when new interviews do not meaningfully add to codes and themes already represented in the previously collected data). Based on previous experience on assessing user preferences, and literature in sampling for qualitative research, we expect to reach saturation for thematic analysis of patients (20–24/country) and professional users, while an exploratory analysis of the decision makers perspectives (overall professionals: 10–15/country, although only 3/country are expected to be decision makers).

Participants are purposively sampled, with a maximum variation approach, which seeks to capture the maximum variation for a defined spectrum to identify information-rich cases. A sampling frame, with potential participants matching the eligibility and purposive sampling criteria, was created based on demographic and trial data. Patients are then invited to participate by the local study staff.

### Data management

All source data remain confidential and are securely stored in designated locations, with restricted access to authorized personnel, in accordance with relevant data privacy regulations. Each participant is allocated a unique pseudonymous identification number, which is consistently employed across the study for all source data.

Accurate documentation of both paper-based and electronic source data, including original records or certified copies of originals, progress notes, screening logs and data obtained from automated instruments is meticulously maintained. The clinical data captured on paper-based Case Report Forms (CRFs) are entered into a database at the respective sites or clinics. The entry process is facilitated through the web-based Clinical Data Management System,OpenClinica. Any alterations or necessary corrections made to the data within Openclinica is documented and tracked in audit trails. To ensure data quality and accuracy, a set of preprogrammed edit and range checks is implemented withing OpenClinica. Additionally, further validation checks are programmed in "R” using data extracts, including electronically received data, for instance, conducting consistency checks across CRFs.

*Operational characteristics:* Pseudonymised Truenat MTB assays results are automatically uploaded to a secure, encrypted server at FIND using a Global SIM card on each Truelab micro-PCR device in the intervention clinic on a regular basis using a File Transfer Protocol (FTP). This data maintains participant’s confidentiality thereby ensuring subject’s anonymity. Laboratory results such as smear microscopy or off-site Xpert MTB/RIF Ultra results are captured by the research team supervisor and recorded in the participants study file. Assay data (rates of non-determined results) and device data (instrument failures) are securely uploaded to the FIND server and accessed by the trial data manager. Instrument maintenance logs will be reviewed at the end of the study, for error, invalid and indeterminate rates.

*Health system cost data:* Excel spreadsheets and word documents with information on health system costs were filled out by each site and stored in the secure OneDrive trial folder. No patient data was captured in these spreadsheets, and this included information such as cost of site finalization, cost of equipment and consumables, training costs, other office and admin costs, and other key costs related to the functioning of the trial at the sites. Site teams were further interviewed on current HIV and TB diagnostic practices at a representative set of study clinics as well as external laboratories or larger health facilities linked to the study clinics. This information was also documented and stored on the restricted-access OneDrive folder.

*User preferences interview data:* interviews are recorded and transcribed in the original language following a standardized procedure. Transcripts are pseudonymized with a participant ID and reviewed against the recording to ensure accuracy of the transcription. Only pseudonymized transcripts, survey answers and notes are kept on the secure trial folder. Destruction of audio-files after transcription is documented. Files are translated to English using a standardized procedure, that include review of the translation by a second researcher, for analysis.

### Data analyses

The effectiveness analyses will be done using the following datasets. The Intent-to treat (ITT) population consists of all randomized participants in the groups to which they were randomly assigned. The Modified Intent-to Treat (MITT) consists of all participants in ITT population who have partial outcomes (i.e., data available for V2 or V3). The Per-Protocol (PP) population consists of all participants who fulfil the protocol in the terms of eligibility, interventions, and outcome assessment without any major protocol deviation. The Per-Protocol Modified (PPM) population consists of all participants in the PP population who were identified to be subjected to the protocol deviation documented in 173/CNBS/23.

*General approach:* All analyses are performed on the MITT and PP populations. Because there was a protocol deviation (i.e., participants with missing V2 or V3), the potential difference in the primary outcome between the two populations will be explored. All analyses (i.e., generalized linear models and proportional hazards models) account for the effect of the clusters using a mixed model approach, introducing a random intercept for each cluster. Details of the models for each outcome are given in the sections below. For all statistical tests, alpha is set to 0.05 for rejecting the null hypothesis. Since multiple comparisons are performed, p-values are adjusted using Benjamini–Hochberg method. The proportion of observations with missing values will be summarised for all outcome variables. Missing and invalid data will not be imputed. Baseline measures will be reported for individuals with missing data on an outcome of interest, including those who withdraw or are lost to follow-up. For each analysis, the baseline measures (demographic and laboratory data) are compared between participants with complete and incomplete data using T-Tests or Chi2 tests, for continuous or categorical variables respectively.

*Analysis of primary outcome:* The analysis for comparing the counts between the control and the intervention arms are done using a GLMM (lme4 package, lmer function in R) with Poisson distribution using log link function, where a cluster will be specified as a random factor (i.e. random intercept) and recruitment time by clinic is specified as a random effect nested within each cluster (i.e. random intercept and random slope). The analysis for comparing the proportions between the control and the intervention arms is done using a GLMM (lme4 package, lmer function in R) with binomial distribution using logit link function, where a cluster will be specified as a random factor (i.e., random intercept). The models are adjusted for gender, age, HIV status, country, and asset index that is described below. In case of necessity, the models may be adjusted for the setting (i.e., urban, rural, peri-rural nesting the clusters), and site. ORs will be estimated by calculating the exponent of the fixed-effect (i.e., arm) coefficients that are extracted from the models described above. 95% confidence intervals will be calculated using Wald method.

*Analysis of secondary outcomes:* The analysis of the binary outcomes are performed in the same way as the analysis of primary outcomes using GLMMs with Poisson or binomial distributions, for count or proportion data respectively. Time-to-event analyses are performed using mixed-effect Cox proportional hazards (PH) regression models (coxme package, coxme function in R). Kaplan Meier curves are used to graphically represent data. The possibility of non-proportional hazards is assessed using Schoenfeld individual test and its graphical representation. In case of non-proportional hazards, we consider splitting the time, using an interaction term, a complementary log–log generalized linear model, or a combination of these approaches. If non-proportional hazard is caused by a confounder, we may set that variable as a strata. As for primary outcome, the models are adjusted for gender, age, HIV status, country, and asset index that is described below. In case of necessity, the models may be adjusted for the setting (i.e., urban, rural, peri-rural nesting the clusters), and site.

*Descriptive statistics:* Tables are generated to summarize the characteristics of the participants in the ITT, MITT, PP and PPM populations. The number of participants included and excluded are reported, overall and for each arm within the hierarchy of each cluster and site. Among the included participants, outcomes of interest as described above as well as covariates such as sex, age (continuous and categorical as age groups defined as: 18–30, 31–40, 41–50, > 50), HIV status, site (i.e., 4 different institutions), country setting (urban, peri-urban rural), asset index and the variables that are used for determining the asset index will be summarized.

*User preferences:* Descriptive analyses are conducted for the survey data (Ease of Use) and the score of the SUS will be estimated [22]. The Frequency of errors captured during direct observations to operators as well as the characteristics of interview participants (e.g., number of patients, healthcare providers, decision makers; respondents from intervention and control clinics, and number of patients positive and negative for TB will be described.

For the user preferences data, thematic analysis is conducted using NVivo software [23]. This begins with familiarisation of the data, followed by coding, generation of initial themes for review, development, until they are defined and named. For the coding process, we use a pre-defined coding framework, which is updated as the analysis is conducted, to reflect emerging themes. The updated Consolidated Framework for Implementation Research is utilized to support the development of the coding framework and presentation of the results of the analysis [24]. This framework was selected after initial familiarization with the data and because it aligns with the research questions. Reporting will follow guidelines for qualitative studies (Consolidated criteria for reporting qualitative research (COREQ), 2007) [25].

*Cost-effectiveness:* Patient costs are estimated as the mean cost per patient evaluated. These costs encompass both direct and indirect costs arising from the process of undergoing diagnostic assessment for TB. Direct costs include all medical (including consultation fees and any out-of- pocket payment for medicines, X-rays, and diagnostics) and non-medical expenses (including travel costs of participants and caregivers, food costs incurred while in hospital, money spent buying any special foods). Indirect costs are estimated as the opportunity cost of time spent seeking care (from the time of symptom onset to the time of treatment initiation), plus any lost productivity due to illness. Additional information from the scientific literature are used to estimate costs that are not directly estimated (e.g., hospitalization costs, costs of TB treatment). We use patient interviews to record the self-reported number of working days lost due to diagnostic assessment for TB, in both arms. This is directly estimated; no additional statistical methods will be used. 95% confidence intervals will be calculated assuming a Poisson distribution. To estimate loss in income, we record the number of missed working days or missed working hours owing to TB illness, from the time of symptom onset to the time of treatment initiation. This number of days/hours is multiplied by the average daily/hourly wage of employed laborers in each country to estimate the monthly earnings lost as a result of seeking care.

We estimate the health system cost per participant tested for TB on-site via the novel Truenat platform/MTB Assays platform versus the hub-and-spoke standard of care, predominantly off-site testing with Xpert MTB/RIF (Cepheid, Inc., Sunnyvale, CA, USA). These include cost for equipment, staffing, consumables, training, communication, and monitoring and evaluation. We estimate ranges for health service delivery costs using trial expense reports, facility assessments, and project staff interviews. We refer to existing scientific literature, information from facility assessments, expense reports, and project staff interviews to estimate the health system cost per patient treated. Our modified societal perspective includes both health system costs and patient costs. Health system costs and patient costs are summed on an individual basis to generate an estimate of the per-patient cost of diagnosis and treatment. This value is estimated as a mean across all participants providing these data. Health system costs are divided by the total number of participants at each site. We report this outcome both by site and by country.

Benefit incidence analysis (BIA) is applied to assess the distribution of benefits of novel Truenat platform/MTB Assays platform (Int 1) compared to the hub-and-spoke standard of care (Int 0) and the centralized approach (Int 2). To conduct the analysis, data on health service utilization and unit costs for each strategy, categorized by socio-economic groups, are estimated. Health service utilization rate is estimated from the trial and expressed as the number and proportion of TB detected and started on treatment across each strategy and stratified by quintile (i.e., which indicates the extent to which the investments made in each diagnostic are allocated equitably, not considering only point of diagnosis, but also returning for test results, treatment initiation and any other needed follow up). The cost incurred by the public sector providers is derived from the data gathered on unit cost of each diagnostic strategy. Regarding the SES status, a simplified asset indices adopted from standardized Equity Tool (https://www.equitytool.org/) is used to collect data on asset ownership (i.e., presence of a refrigerator, a mobile phone, a television, electricity, type of toilet, water supply, cooking fuel, and materials of the wall and floor) of study participants and a standardized scoring of these questions are used to assign a wealth quintile for each respondents. Then health service utilization rate is then multiplied by the unit costs of each diagnostic Intervention. The benefits of each diagnostic strategy utilization will be aggregated and expressed in monetary terms. Concentration curves and indexes are computed to illustrate the extent of inequality in the distribution of health spending across quintiles.

### Monitoring

TB-CAPT has implemented a risk-based monitoring approach to make best use of resources and to focus on site monitoring activities to sites which pose highest risk defined as high query rates and low enrolment. Three to five participants per clinic are selected randomly by the monitor, among participants enrolled since the previous visit. 100% source data review and database verification, for key variables, are performed on these participants. For any participant raising more than 5 queries, additional participants are selected randomly by the monitor, for additional review. All monitoring findings are communicated to the site PI for resolution.

The rationale behind this monitoring strategy is based on the fact that this study is being coordinated by experienced clinical trial sites, located throughout Mozambique, and Tanzania.

A combination of centralized, off-site (remote) and on-site monitoring have been conducted for this study, described in detail below:

All sites had an initial on-site visit (Site Initiation Visit; SIV), lasting at least 2 days, and was conducted by the FIND Trial manager and Trial Monitor. All sites had a first interim/on-site visit after recruiting 25% of the participants, and a second interim/on-site visit after recruiting 50% of the participants. A two-step close-out approach is taken with remote pre-close out, followed by on-site close-out visits at all sites.

Centralized database monitoring is led by the data management team at LMU, with support from FIND. Regular data checks to OpenClinica are run quarterly by the Trial Data Manager at LMU to check for data discrepancies and queries. Automated validation checks built into the data management system are used for comparing results among tests: Truenat assay runs on the Truenat platform (investigational product), conventional diagnostics (i.e. on-site smear microscopy or referral for Ultra testing).The FIND Data Management team with the support of the Project Manager review reports generated by the by the Trial Data Manager at LMU, such as data listings and queries quarterly to determine if there are any systematic errors, trends or outliers that need further investigation and follow up with the trial site(s).

The FIND Project Management team and the steering group leads conduct centralized monitoring of sample flow and process, site randomization and intervention allocation procedure, testing procedures and use/storage and stock of supplies on a quarterly basis.

### Confidentiality of personal data

All study records and samples are managed in a way that prioritizes participant confidentiality, securely stored with restricted access, while ensuring continuous clinical care provision. The only identifying information present on CRFs is the PID, along with participants sex and date of birth. Usage of the study participants’ data adheres to the terms specified in the informed consent form and complies with applicable data privacy regulations. Participant records may be reviewed by inspectors of regulatory authorities or ethics committees, study monitors and auditors, who are responsible for study quality. An individual’s study data will not be disclosed without participants written consent, except as necessary for monitoring and auditing by the sponsor or its representative, regulatory authorities, or ethics committees, or in case of medical emergencies when written consent cannot be obtained, as deemed in the participant’s best interest by the investigator.

### Ethics and dissemination

TB-CAPT CORE trial has been approved by regulatory and ethical committees in Mozambique (National IRB approval #131/CNBS/22), Tanzania (National IRB approval #NIMR/HQ/R.8c/Vol.I/2323 and TMDA approval #BD.59/62/46/05), and Germany (UKHD S-616/2021). Consent is informed and voluntary, and confidentiality of participants is maintained throughout. Potential participants are asked for written informed consent prior to enrollment into the study. In case of illiteracy, the participant is asked to give its consent by fingerprint while an adult impartial, literate witness present during the entire consent procedure signs the consent to confirm presence during the entire process. All participants have the right to withdraw from the study at any time. Study findings will be presented at scientific conferences and published in peer-reviewed international journals.

## Discussion

The TB-CAPT CORE study is a pragmatic cluster randomized controlled trial, assessing the implementation of Truenat platform/MTB assays at the microscopy center level, in Mozambique and Tanzania, leveraging existing infrastructure around smear microscopy to increase access for patients and transform health systems. Implementation of this promising technology, deployed at the lower levels of the healthcare system, has been carefully planned to ensure integration within the existing diagnostics system, to provide linkage to treatment and to match patient pathways. The trial described here has been planned for optimal integration into the diagnostic network to maximize impact on patient outcomes (time to diagnosis, treatment, cure rates, mortality), support national policy and lay the groundwork for accelerated scale-up in early adopter countries.

If implemented successfully, the testing strategy proposed has the potential to improve patient outcomes, through enabling diagnosis and care closer to the patients. This will prevent transmission through early diagnosisiolation, and treatment initiation when patients first present to the health care system; prevent inadequate treatment by facilitating early diagnosis of drug resistant TB; and prevent deaths through rapid diagnosis and therapy initiation in severely ill patients. Beyond the direct impact of improving TB diagnosis and treatment within the trial countries, the project will have far-reaching positive impact on policy, infrastructure, and human capacity on the African sub-continent.

### Supplementary Information

**Additional file 1.** 

## Data Availability

Anonymized data will be uploaded to a publicly available, scientific data repository, where a DOI will be associated with the dataset.

## Abbreviations

MTB: Mycobacterium tuberculosis
TB-CAPT: The Tuberculosis Close the Gap, Increase Access, and Provide Adequate Therapy Consortium
TB: Tuberculosis
MDR: Multi-drug Resistant
Ultra: Xpert MTB/RIF Ultra
WHO: World Health Organization
MTBC: Mycobacterium tuberculosis Complex
SUS: System Usability Scale
FTP: File Transfer Protocol
ITT: Intent-To-Treat
MITT: Modified Intent-To-Treat
PP: Per Protocol
PPM: Per Protocol Modified
HIV: Human Immunodeficiency Virus
COREQ: Consolidated Criteria for Reporting Qualitative Research
SIV: Site Initiation Visit
