# Supplementary material for: Study protocol: a pragmatic, cluster-randomized controlled trial to evaluate the effect of implementation of the Truenat platform/MTB assays at primary health care clinics in Mozambique and Tanzania (TB-CAPT CORE)
Source: BMC Infect Dis. 2024 Jan 19;24:107. doi: 10.1186/s12879-023-08876-8 (PMC10797907; doi:10.1186/s12879-023-08876-8)
Supplement: Supplementary file 1 — Additional file 1. [file 12879_2023_8876_MOESM1_ESM.docx]

TB-CAPT Consortium

Vinzeigh Leukes^1^, Adam Penn-Nicholson^1^, Morten Ruhwald^1^, Berra Erkosar^1^, Samuel Schumacher^1^, Sunita Singh^1^, Bernard Kivuma^2^, Muhuminu Nuru^2^, Judith Mlenge^2^, Neema Shija^2^, Deogratias Bulime^2^, Dorcas Mnzava^2^, Petro Sabuni^2^, Hosiana Temba^2^, Jamali Siru^2^, Jerry Hella^2^, Jonathan Msafiri^2^, Maja Weisser^2^, Mohamed Mbaruku^2^, Mohamed Sasamalo^2^, Alice Leonard^2^, Ambilikile Malango^2^, Annastazia Alexander^2^, Faith Komakoma^2^, Gloria Msigala^2^, Kasmir Johaness^2^, Grace Mhalu^2^, Mwajabu Hamis^2^, Priscilla Mlay^2^, Robert Ndege^2^, Sera Barasa^2^, Swalehe Masoud^2^, Theonestina Byakuzana^2,^, Anange Lwilla^3^, Benedict Kayombo^3^, Chacha Mangu^3^, Christina Manyama^3^, Theodora Mbunda^3^, Elimina Siyame^3^, Issa Sabi^3^, Last Mwaipopo^3^, Nyanda Elias Ntinginya^3^, Raphael Edom^3^, Willyhelmina Olomi^3^, Delio Elisio^4^, Dinis Nguenha^4^, Edson Mambuque^4^, Joaquim Cossa^4^, Marta Cossa^4^, Neide Gomes^4^, Patricia Manjate^4^, Shilzia Munguambe^4^, Sozinho Acacio^4^, Belen Saavedra^4^, Helio Chiconela^4^, Katia Ribeiro^4^, António Machiana^5^, Bindiya Meggi^5^, Candido Azize Junior^5^, Carla Madeira^5^, Celso Khosa^5^, Claudio Bila^5^, Denise Floripes^5^, Diosdélio Malamule^5^, Sofia Viegas^5^, Albero Garcia-Basteiro^4,6^, Belén Saavedra^6^, Carole Amroune^6^, Joanna Ehrlich^6^, Laura de la Torre Pérez^6^, Sergi Sanz^6^, Friedrich Riess^7^, Katharina Kranzer^7,12^, Michael Hoelscher^7,18,19^, Norbert Heinrich^7,18,19^, Sarah Mutuku^7^, Tejaswi Appalarowthu^7^, Leyla Larson^7^, Maria del Mar Castro Noriega^8^, Claudia Denkinger^8^, Saima Arif^8^, Daniela Maria Cirillo^9^, Elisa Tagliani^9^, Federico Di Marco^9^, Virginia Batignani^9^, Akash Malhotra^10^, David Dowdy^10^, Claudia Schacht^11^, Julia Buech^11^, Caroline Stöhr^11^, Marguerite Massinga Loembé^13^, Pascale Ondoa^13^, Nqobile Ndlovu^13^, Fumbani Brown^13^, Yonas Ghebrekristos^14^, Cindy Hayes^14^, Ilse vanderwalt^14^, Shareef Abrahams^14^, Puleng Marokane^14^, Mbuti Radebe^14^, Neil Martinson^14^, Anura David^15^,Lesley Scott^15^, Lucky Ngwenya^15^, Pedro Da Silva^15^, Riffat Munir^15^, Wendy Stevens^15^ , Charles Abongomera^16^, Klaus Reither^16^, Leon Stieger^16^, Adrian Brink^17^, Chad Centner^17^, Helen Cox^17^, Judi van Heerden^17^, Mark Nicol^17^, Nchimunya Hapeela^17^, Parveen Brown^17^, Reyhana Solomon^17^, Widaad Zemanay^17^, Tania Dolby^17^.

^1^FIND, Geneva, Switzerland

^2^Ifakara Health Institute, Dar es Salaam, Tanzania

^3^Mbeya Medical Research Centre, National Institute for Medical Research (NIMR), Mbeya, Tanzania

^4^Centro de Investigação em Saúde de Manhiça (CISM) Manhica, Mozambique

^5^Instituto Nacional de Saúde (INS), Marracuene, Mozambique

^6^ISGlobal, Hospital Clínic – Universitat de Barcelona, Barcelona, Spain.

^7^Division of Infectious Diseases and Tropical Medicine, LMU University Hospital, LMU Munich

^8^Division of Infectious Disease and Tropical Medicine, Heidelberg University Hospital, Heidelberg, Germany

^9^Emerging Bacterial Pathogens Unit, IRCCS San Raffaele Scientific Institute, Milan, Italy

^10^Johns Hopkins University, Baltimore, Maryland, USA

^11^LINQ Management, Berlin, Germany

^12^Clinical Research Department, London School of Hygiene and Tropical Medicine, London, UK.

^13^African Society for Laboratory Medicine, Addis Ababa, Ethiopia

^14^National Health Laboratory Service, Johannesburg, South Africa

^15^WITS Health Consortium, Johannesburg, South Africa

^16^Swiss Tropical and Public Health Institute, Allschwil, Switzerland

^17^Division of Medical Microbiology, University of Cape Town, South Africa

^18^Fraunhofer Institute for Translational Medicine and Pharmacology ITMP; Immunology, Infection and Pandemic Research, Munich, Germany

^19^Unit Global Health, Helmholtz Zentrum München, German Research Center for Environmental Health (HMGU), Neuherberg, Germany
